# Development and application of a blocking ELISA method based on Cap protein for detecting antibodies against porcine circovirus 2

**DOI:** 10.1128/spectrum.03040-24

**Published:** 2025-03-31

**Authors:** Qingyan Liu, Shuolei Gao, Jiannan Li, Jingyi Yang, Yuxiang Zhu, Jun Zhu, YanJun Zhou, TongLing Shan, Wu Tong, Hao Zheng, Ning Kong, YiFeng Jiang, ChangLong Liu, GuangZhi Tong, Hai Yu

**Affiliations:** 1 Shanghai Veterinary Research Institute, Chinese Academy of Agricultural Sciences 118161 , Shanghai, Shanghai, China; 2 Tianjin Agricultural University 91633, Tianjin, Tianjin, China; 3 Jiangsu Co-Innovation Center for Prevention and Control of Important Animal Infectious Diseases and Zoonoses, Yangzhou, China; University of Prince Edward Island, Charlottetown, Prince Edward Island, Canada

**Keywords:** porcine circovirus type 2 (PCV2), Cap protein monoclonal antibody, blocking ELISA

## Abstract

Porcine circovirus type 2 (PCV2) serves as the key pathogen linked to porcine circovirus-associated disease, representing a considerable risk to the worldwide swine industry. A blocking enzyme-linked immunosorbent assay (ELISA) was established to identify antibodies specifically targeting PCV2, employing recombinant Cap protein as the antigen and a monoclonal antibody against PCV2 Cap protein as the detector antibody. Utilizing receiver operating characteristic curve analysis, a cutoff value of 33.8% was determined to distinguish between positive and negative serum samples. The sensitivity and specificity of this blocking ELISA method were reported at 94.7% and 96.1%, respectively. Notably, this approach exclusively identified antibodies for PCV2, showing no cross-reactivity with antibodies related to African swine fever virus (ASFV), Porcine epidemic diarrhea virus (PEDV), Porcine pseudorabies virus (PRV), and porcine reproductive and respiratory syndrome virus. Both intra-assay and inter-assay coefficients of variation were less than 10%. In a comparison involving 402 porcine serum samples, the agreement rate with a commercial indirect ELISA kit reached 98.76%, with a kappa value of 0.888, reflecting high concordance between the two testing methods. This study showcases the blocking ELISA method as an efficient and standardized approach for serological monitoring of PCV2 in swine populations and for assessing seroconversion in vaccinated pigs.

**IMPORTANCE:**

Porcine circovirus type 2 (PCV2) has become recognized as a pathogen of significant economic concern within the swine industry. PCV2 mainly affects the immune systems of pigs, leading to a reduction in lymphocytes and resulting in immune suppression in the affected animals. Co-infection with other porcine pathogens can enhance PCV2 infection and exacerbate porcine circovirus disease. Currently, the kits available for detecting PCV2 antibodies primarily employ indirect enzyme-linked immunosorbent assay (ELISA); however, this method is prone to false positives. In contrast, the blocking ELISA method offers enhanced specificity and provides a more straightforward interpretation of results. Previous studies utilizing blocking ELISA for PCV2 antibody detection have depended on plates coated with purified PCV2 virus, a process that is both technically challenging and time-consuming. Consequently, there is a pressing need to develop a new blocking ELISA method that is more efficient to detect antibodies against PCV2.

## INTRODUCTION

Porcine circovirus type 2 (PCV2) was first identified in 1998 (1) and has since become a major pathogen for swine, leading to considerable economic challenges for the global pig farming sector. The initial isolation of PCV2 occurred in Canada, where it was extracted from pigs exhibiting symptoms of postweaning multisystemic wasting syndrome (PMWS) (1, 2). This notable discovery highlighted the importance of PCV2 in swine health and management. Further research has indicated that PCV2 contributes significantly to a range of reproductive disorders, impacting breeding success and overall herd productivity (3). Additionally, the virus is implicated in porcine dermatitis and nephropathy syndrome (PDNS), a condition characterized by skin lesions and kidney issues in infected pigs (4). PCV2 is also involved in the porcine respiratory disease complex (PRDC), which encompasses a variety of respiratory illnesses that can affect pigs (5). Furthermore, it is associated with proliferative and necrotizing pneumonia, a severe lung condition that can lead to increased morbidity and mortality (6). In the United States, the disease caused by this pathogen is commonly referred to as porcine circovirus-associated disease, whereas in Europe, it is generally known as porcine circovirus disease (PCVD). These differing terminologies reflect regional variations in the understanding and categorization of the disease, yet they all highlight the significant effects of PCV2 on swine health across the globe (7, 8).

PCV2 belongs to the Circoviridae family and is classified within the *Circovirus* genus. As the smallest known DNA virus that infects mammals, PCV2 features a single-stranded, non-enveloped circular genome consisting of 1,766 to 1,768 nucleotides (9, 10). Research shows that PCV2 comprises 60 capsid subunits, which are arranged into 12 pentamers, forming an icosahedral virus particle (11). Notably, PCV2 exhibits ongoing evolution under selective pressures, resulting in the emergence of increasingly pathogenic genotypes over time (12). Currently, nine distinct genotypes of PCV2 have been recognized (a–i), with the PCV2b genotype being the most widely distributed globally, while PCV2d has emerged as the dominant strain amid the current global transition of PCV2 genotypes (13–15). Similar to PCV1, PCV2 harbors 11 open reading frames (ORFs), with ORF1 and ORF2 being the two major frames. Research suggests that ORF1 encodes two replicase proteins, whereas ORF2 is associated with the viral structural capsid protein (16). The capsid protein, approximately 30 kDa in size, is the sole structural protein of PCV2 and possesses antigenic epitopes capable (17) of triggering robust immune responses in animals infected with PCV2 (18). Consequently, the capsid protein is considered a prime candidate for the creation of serodiagnostic assays and vaccines (9).

To date, various methods have been employed to detect antibodies against PCV2 in serum, with the immunoperoxidase monolayer assay (IPMA) (19, 20) and the indirect immunofluorescence assay (IFA) being the most commonly used methods (21). However, these techniques are highly technical, time-consuming, and require laboratory settings. In contrast, enzyme-linked immunosorbent assay (ELISA) methods are favored for their simplicity, rapidity, and cost-effectiveness, making them the predominant assay techniques for scientific research, disease diagnosis, and evaluation of vaccine immune efficacy (22). Increasing research has unveiled four distinct ELISA methods suitable for the detection of PCV2 antibodies in either serum or fecal samples: indirect ELISA (23–25), competitive ELISA (26–28), double-antigen sandwich ELISA (29, 30), and blocking ELISA (31). While commercial kits for detecting PCV2 antibodies primarily utilize indirect ELISA, this method is susceptible to false positives. In contrast, blocking ELISA exhibits higher specificity than indirect ELISA, and the interpretation of results is more straightforward.

In this study, after immunizing BALB/c mice with the rescued virus rPCV2d, one monoclonal antibody (mAb) 3G5 demonstrated a blocking effect. Consequently, this discovery led to the development of a blocking ELISA method that utilized mAb 3G5 as the detection antibody. The resulting assay was validated for its high sensitivity and specificity in the detection of PCV2 antibodies, thereby establishing it as a novel and effective tool for the surveillance of PCV2.

## RESULTS

### Evolutionary tree analysis of PCV2d and rescue of recombinant virus rPCV2d

**Fig 1:**
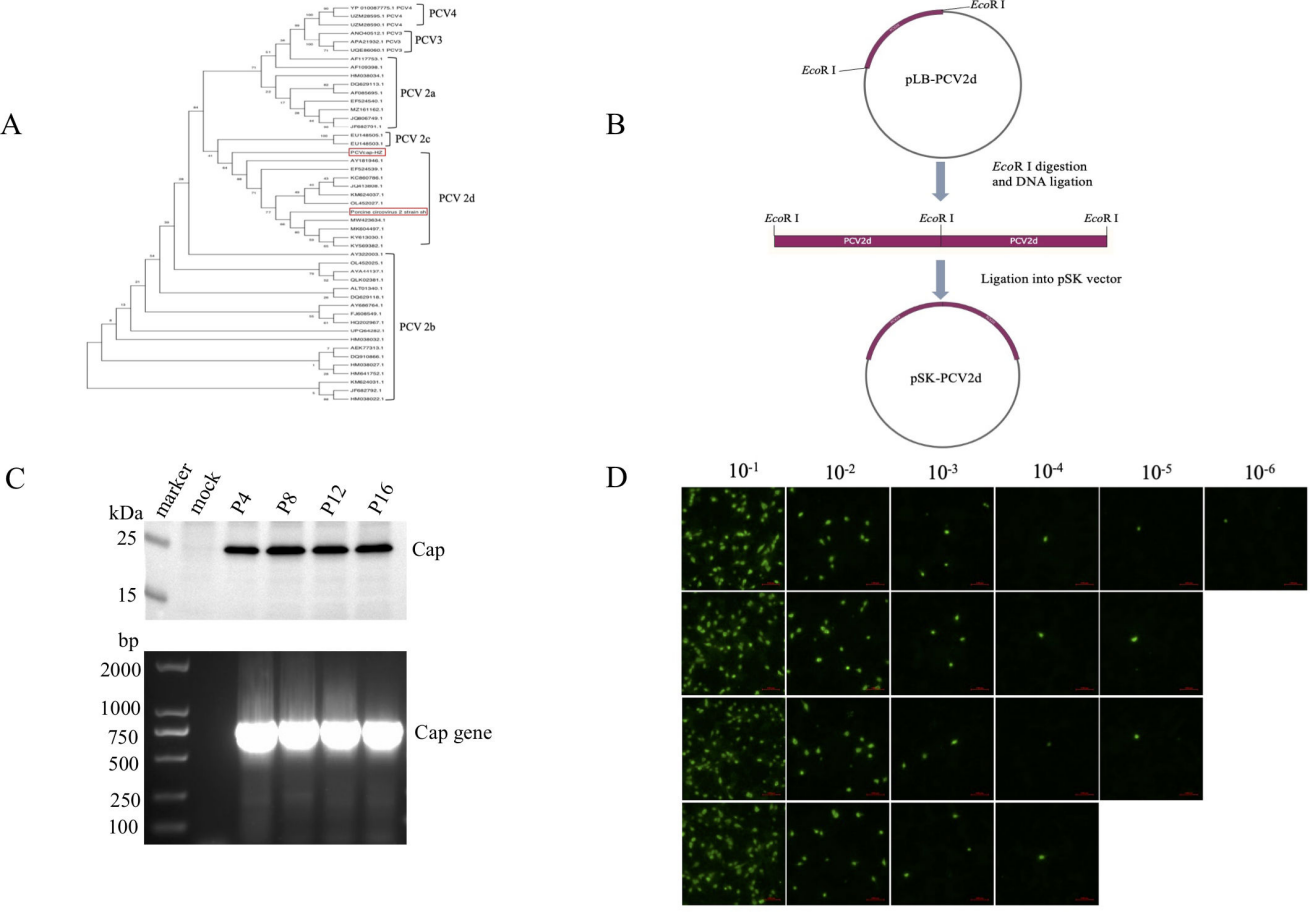
The evolutionary tree analysis of PCV2d and the rescue of the recombinant virus rPCV2d. (A) The entire genome of PCV2 was analyzed using Mega software. (B) The PCV2d genome was digested with the *Eco*R I restriction enzyme and subsequently purified. The purified PCV2d genomic DNA was then ligated with T4 DNA ligase to produce tandem dimers. These tandem dimers were separated using 1% agarose gel electrophoresis and ligated with the pSK vector (predigested with *Eco*R I restriction enzyme) to obtain the recombinant plasmid pSK-PCV2d. This plasmid was transfected into PK-15 cells to rescue the recombinant virus rPCV2d. (C) PCR analysis was performed on the recombinant virus rPCV2d of P4, P8, P12, and P16 generation. The cell culture supernatant underwent DNA extraction and PCR using primers targeting the PCV2d Cap gene. Additionally, Western blot analysis of the recombinant virus rPCV2d of P4, P8, P12, and P16 generation was conducted, with the PK-15 cell culture supernatant being detected using a polyclonal antibody against the PCV2d Cap protein. (D) The TCID_50_ of rescued virus rPCV2d was measured in PK-15 cells with four repeats conducted for each dilution titer.

Phylogenetic tree analysis of the full-length genome sequence indicates that the isolated virus PCV2 belongs to the 2d subtype(Fig. 1A). The purified recombinant plasmid pSK-PCV2d was transfected into PK-15 cells and incubated for 72 hours to generate the P0 generation recombinant virus, designated rPCV2d. Subsequently, the rPCV2d was obtained through propagating on PK-15 cells for 16 generations, with the cells undergoing three cycles of freezing and thawing. PCR and Western blot experiments confirmed the successful rescue of the recombinant virus rPCV2d(Fig. 1C). To verify the genomic stability of rPCV2d, the P4, P8, P12, and P16 cell culture supernatants containing rPCV2d were subjected to DNA extraction, followed by full-length DNA sequencing. The sequences from the P4, P8, P12, and P16 generation of rPCV2d virus were aligned with the original PCV2d sequences. No mutations were observed among these four generations of the virus (data not shown). These results demonstrate that the rPCV2d genome remains stable across 16 generations. Additionally, the titer of the recombinant virus was measured at 10^5.5^ TCID_50_/mL(Fig. 1D),which meets the experimental criteria.

### Preparation and purification of mAb against PCV2 Cap

**Fig 2:**
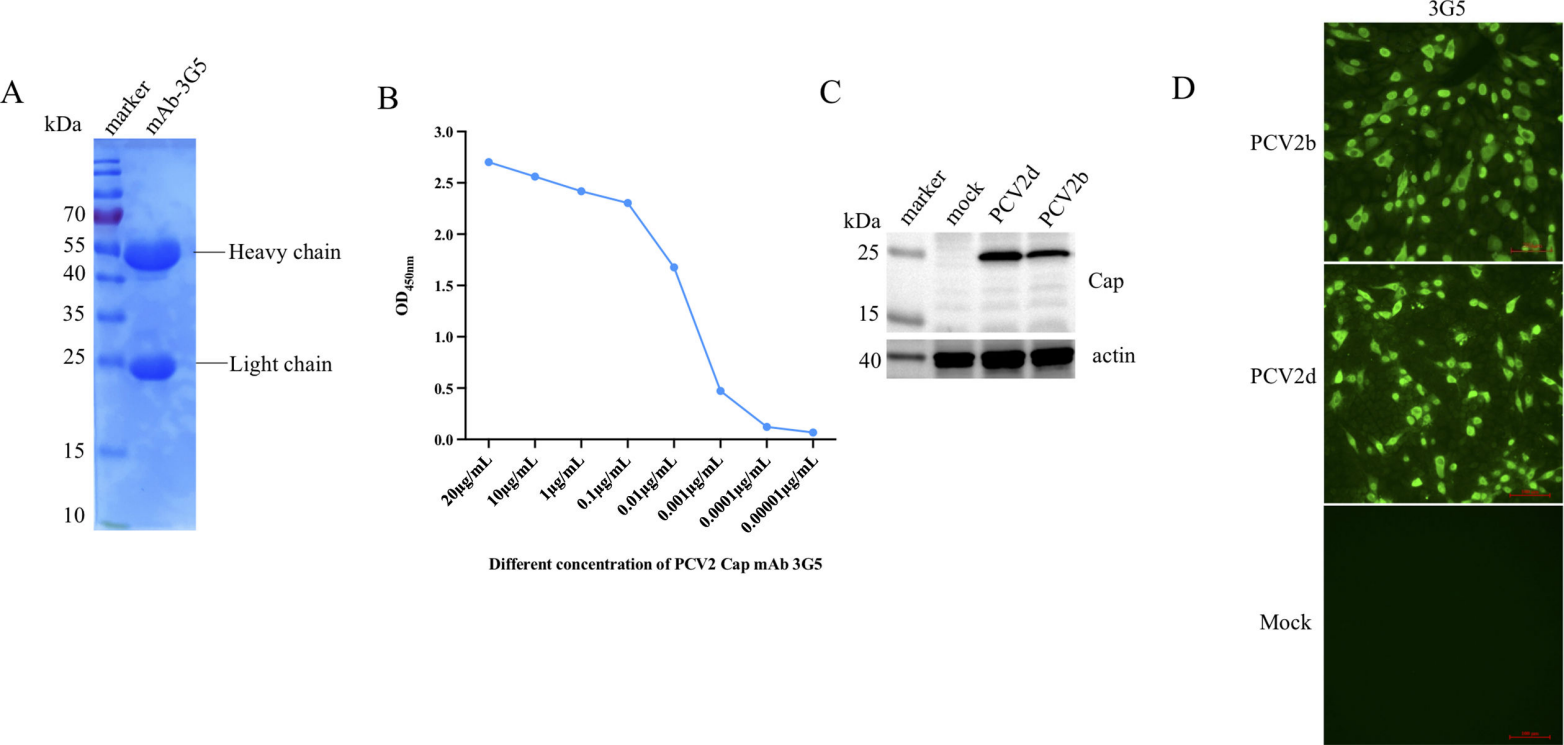
Purification and identification of mAb 3G5 against PCV2. (A) SDS-PAGE analysis confirmed the purity of mAb 3G5. (B) ELISA analysis demonstrated the binding affinity of mAb 3G5 to the PCV2 Cap protein. (C) Western blot was employed to verify the presence of PCV2b and rPCV2d strains in PK-15 cells infected with these viruses, using purified mAb 3G5. (D) IFA analysis further validated the binding of mAb 3G5 to PK-15 cells infected with PCV2b and rPCV2d, following incubation with mAb 3G5 and 488-conjugated goat anti-mouse IgG (H + L).

Monoclonal antibodies were generated using traditional methods (32). A hybridoma cell line, designated mAb 3G5, was established following multiple rounds of screening and subcloning. Mouse ascites were prepared and purified using a protein A gravity-flow column kit, resulting in the acquisition of the mAb 3G5. The SDS-PAGE experiment confirmed that the purified mAb 3G5 exhibited a clear band at 50 kDa and 25 kDa, corresponding to the heavy and light chains of the antibody, respectively (Fig. 2A). Further examination using an ELISA method established that the purified mAb 3G5 had an affinity for the Cap protein, as depicted in Fig. 2B. As illustrated in Fig. 2C, purified mAb 3G5 exhibited specific reactivity with the Cap protein in PK-15 cells infected with PCV2, as evidenced by Western blot analysis. Notably, strong green fluorescence was observed in PK-15 cells that were infected with PCV2 during IFA, whereas PK-15 cells that did not undergo infection displayed no detectable green fluorescence. This contrasting result underscores the specificity of mAb 3G5 in recognizing the Cap protein associated with PCV2 (Fig. 2D).

### Development of the blocking ELISA method based on PCV2 Cap protein and mAb 3G5

**Fig 3:**
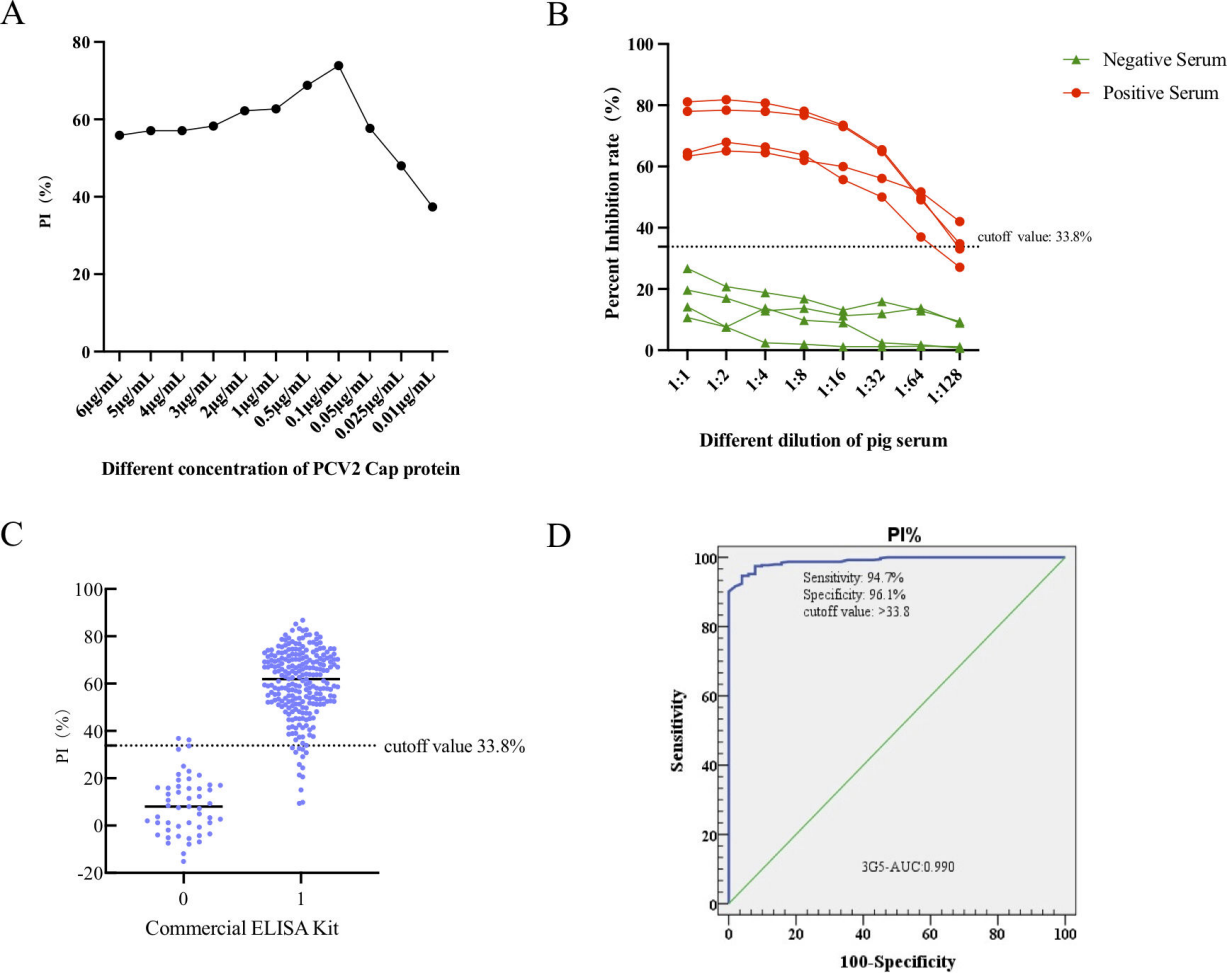
Optimization of the blocking ELISA and ROC analysis of positive and negative serum samples’ critical values. (A) Determination of optimal coating concentration of PCV2 Cap protein. (B) Determination of the optimal dilution concentration of the serum to be tested. (C) Interactive dot diagram analysis of the 3G5-based blocking ELISA showing the cutoff value of serum samples was set to 33.8%. (D) The ROC analysis of the blocking ELISA demonstrated an area under the curve (AUC) of 0.990 (95% confidence interval: 0.989–1.0).

The purified mAb 3G5 was labeled with horseradish peroxidase (HRP) and utilized in a blocking ELISA. The optimal coating concentration of the Cap protein was established at 0.1 µg/mL using the checkerboard method (Fig. 3A), while the optimal dilution for mAb 3G5 with HRP conjugated was determined to be 1:2,000 (Fig. 3B). Furthermore, a serum dilution of 1:2 was found to be optimal. These conditions were consistently applied in subsequent blocking ELISA experiments. In total, 51 serum samples negative for PCV2 and 395 positive samples were examined in order to determine the cutoff value for the blocking ELISA method. Each of these samples underwent the blocking ELISA test, from which the percent inhibition (PI) for each sample was computed. As illustrated in Fig. 3C, the diagnostic specificity and sensitivity were found to be 96.1% and 94.7%, respectively, with a PI cutoff value of 33.8%. According to the receiver operating characteristic (ROC) analysis, the area under the curve (AUC) was 0.990 (95% confidence interval: 0.989–1.0), indicating that the blocking ELISA possesses high accuracy (Fig. 3D). Consequently, serum samples were classified as positive when the PI exceeded 33.8%, whereas samples with a PI below this threshold were deemed negative.

### Sensitivity and specificity of the blocking ELISA

**Fig 4:**
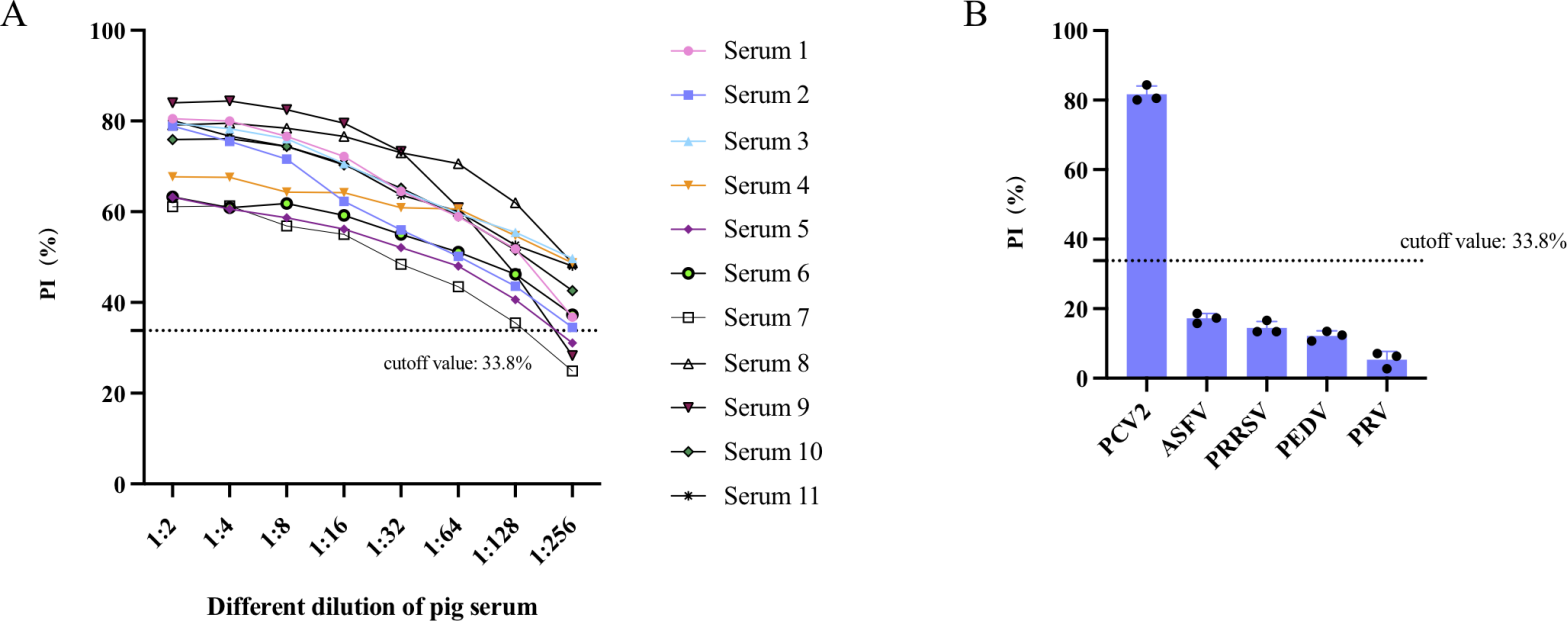
Sensitivity and specificity analysis of the developed blocking ELISA. (A) Analytical sensitivity of the blocking ELISA. (B) Evaluation specificity of the blocking ELISA by detecting swine positive serum for five viruses, including PCV2, ASFV, PRRSV, PEDV, and PRV. PRRSV, porcine reproductive and respiratory syndrome virus.

To evaluate the sensitivity of the method employed in this study, a total of 11 serum samples were systematically assessed. This assessment involved performing twofold serial dilutions of a serum sample known to be positive for PCV2, with dilution ratios ranging from 1:2 to 1:256. The results, illustrated in Fig. 4A, indicate that at a dilution level of 1:128, the PI of all samples exceeded 33.8%. Notably, the dilution of eight serum samples reached up to 1:256, indicating that the blocking ELISA method exhibits good analytical sensitivity.

To test the specificity of the newly established blocking ELISA, various serum samples were analyzed for reactivity against other swine-infecting viruses. As illustrated in Fig. 4B, only the PCV2 antiserum tested positive, while sera against other swine-infecting viruses, such as ASFV, PEDV, PRV, and porcine reproductive and respiratory syndrome virus (PRRSV), showed negative results with lower blocking values compared to the cutoff value. The PI value of the anti-PCV2 serum was the highest, reaching 84%.

### Repeatability and reproducibility of the blocking ELISA

To assess the repeatability and reproducibility of the newly developed blocking ELISA, seven PCV2-positive serum samples and four PCV2-negative serum samples were tested using this method to calculate the coefficient of variation (CV) for both intra-assay and inter-assay precision. According to the data presented in Table 1, the intra-assay CVs for the PI displayed a range from 0.2% to 9.3%. Similarly, the inter-assay CVs exhibited a range from 0.1% to 9.2%. Both sets of CV values were below 10%, suggesting that the newly developed blocking ELISA demonstrates good repeatability and reproducibility.

**TABLE 1:** Intra-assay and inter-assay of the blocking ELISA

Samples	Intra-assay			Inter-assay		
	Mean PI (%)	SD	CV (%)	Mean PI (%)	SD	CV (%)
1	81.3	0.009788	1.2	83.4	0.013777	1.7
2	78.6	0.017194	2.2	67.8	0.000686	0.1
3	67.7	0.001674	0.2	59.8	0.013312	2.2
4	75.4	0.03654	4.8	72.6	0.023986	3.3
5	84.0	0.004422	0.5	70.3	0.005787	0.8
6	79.2	0.002786	0.4	79.4	0.004493	0.6
7	75.4	0.009353	1.2	72.5	0.02521	3.5
8	22.5	0.011031	4.9	21.4	0.014584	6.8
9	19.4	0.005508	2.8	16.7	0.015428	9.2
10	20.2	0.005292	2.6	19.0	0.017444	9.2
11	4.3	0.004	9.3	4.0	0.003541	8.9

### Comparison of the blocking ELISA and commercial indirect ELISA kit

In order to assess the applicability of the developed blocking ELISA for detecting clinical serum samples, a total of 402 pig serum samples from three large-scale farms located in Zhejiang province were tested using the blocking ELISA and a commercial indirect ELISA antibody kit. As presented in Table 2, the positive rate of PCV2 antibodies detected by the commercial indirect ELISA kit was 94.78% (381/402), while the developed blocking ELISA yielded a positive rate of 93.53% (376/402). The agreement rate between the two assays was 98.76%. Statistical analysis revealed a kappa value of 0.888, indicating a strong agreement between the commercial indirect ELISA kit and the blocking ELISA. The results indicate that the developed blocking ELISA utilizing mAb 3G5 shows a strong agreement with the available commercial indirect ELISA kit.

**TABLE 2:** Comparisons of the blocking ELISA and commercial indirect ELISA kit by detecting field serum samples^*a*^

	Serum samples	Blocking ELISA			
		+	−	Total	Agreement
Commercial kit	+	376	5	381	
	−	0	21	21	
	Total	376	26	402	98.76%
	Kappa value			0.888	

^*a*^ "+"denotes the number of positive samples, and "−"represents the number of negative samples.

## DISCUSSION

PCV2 has emerged as a pathogen of considerable economic importance within the swine industry. PCV2 primarily targets the immune system of pigs, resulting in lymphocyte depletion and subsequent immune suppression in infected animals (33). Research has shown that piglets that are solely infected with PCV2 tend to show only mild clinical signs (34, 35). However, co-infection with other porcine pathogens, such as swine influenza virus (36), porcine parvovirus (37–39), PRRSV (40, 41), porcine hokovirus (42), classical swine fever virus (43), *Mycoplasma hyopneumoniae* (44), and *Salmonella* spp. (45, 46), can enhance PCV2 infection and exacerbate PCVD. Additionally, factors such as immunostimulation from adjuvants, vaccination failures, and stress can further aggravate PCVD (8). Notable conditions associated with PCVD include PMWS (47), PDNS (48), PRDC, PCV2 reproductive disorders, proliferative and necrotizing pneumonia, and neonatal congenital tremor (7, 49). Since its discovery in 1998, PCV2 and PCVD have caused substantial economic challenges for the global swine industry (50). Although several PCV2 vaccines are available on the market, these vaccines typically target a single PCV2 genotype (51, 52). Given that PCV2 exhibits the highest evolutionary rate when compared to other DNA viruses, the continuous emergence and prevalence of new genotypes present ongoing challenges in vaccine development (52). Consequently, monitoring PCV2 antibody levels in pigs is crucial for formulating effective immunization programs and evaluating vaccine efficacy.

PCV2 antibodies in pigs can be generated through natural infection, the transmission of maternal immunity from sows to piglets, or vaccination. The application of serological methods for the detection of PCV2 antibodies plays a fundamental role in monitoring the prevalence of this virus within swine herds. Common diagnostic assays for detecting PCV2 antibodies include the IFA, IPMA, and ELISA (28, 53). However, both IPMA and IFA detection methods necessitate an aseptic environment, and their experimental processes are complex, often taking more than 72 hours. Serum neutralization tests are also time-consuming and require advanced technical expertise, rendering them unsuitable for large-scale serological monitoring (54). Currently, ELISA is the most widely employed immunological detection technology, offering significant advantages in sensitivity compared to other methods. It encompasses various formats, including indirect ELISA, competitive ELISA, double-antibody sandwich ELISA, and blocking ELISA, all of which are suitable for testing a large number of serum samples (55). Huang et al. established a blocking ELISA method utilizing mAb 1D2 which can detect neutralizing antibodies against PCV2 in serum (31). However, this assay faces challenges, as the serum coating the ELISA plate can exhibit uneven antibody levels across different batches, and it requires purified PCV2 virus, which entails a heavy workload and is time-consuming. In this study, we employed the Cap protein produced through the baculovirus expression system as the antigen for coating and mAb 3G5 tagged with HRP as the secondary antibody. The resulting blocking ELISA method demonstrates high sensitivity and specificity, with a reduced processing time compared to previous blocking ELISA methods.

In this study, the mAb 3G5 was generated by immunizing mice with purified rPCV2d virus. Additionally, prokaryotically expressed PCV2 Cap protein was also evaluated as a coating antigen; however, it demonstrated lower specificity and blocking rates (data not shown). This suggests that eukaryotically expressed proteins may better resemble the Cap protein of the natural virus in structure and exhibit enhanced reactivity with the PCV2 antibody. Furthermore, it was confirmed that 3G5 can bind to both PCV2b and rPCV2d genotypes through IFA and Western blot analysis. Consequently, the blocking ELISA based on mAb 3G5 can be employed to assess antibody levels against these PCV2 strains. Additionally, mAb 3G5 labeled with HRP was utilized in the blocking ELISA method to minimize false positive results, thereby streamlining and expediting the procedure. To evaluate the practical application of the established blocking ELISA method, we conducted a comparison with a commercial indirect ELISA kit. A total of 402 serum samples sourced from three different commercial farms were tested using both the blocking ELISA based on mAb 3G5 and the commercial indirect ELISA kit. The comparative results revealed an impressive coincidence rate of 98.76% between the outcomes generated by the blocking ELISA and those obtained from the commercial indirect ELISA kit. The blocking ELISA method demonstrated high consistency with commercially available kits for detecting serum samples from field farms (kappa = 0.888). Such findings underscore the reliability of the blocking ELISA method, suggesting that it can serve as an effective tool for monitoring the efficacy of the PCV2 vaccine in field settings.

In summary, this study demonstrated that the blocking ELISA utilizing mAb 3G5 is an effective tool for measuring antibody levels against PCV2 in serum samples. It exhibits no cross-reactivity with serum from other viruses and shows a 98.76% consistency rate with commercial indirect ELISA kits. Additionally, a significant positive correlation was observed between the outcomes of the blocking ELISA and the indirect ELISA, reinforcing the validity of the findings. The preliminary applications of this novel blocking ELISA suggest its potential as a time-efficient and standardized method for serological detection of PCV2. Moreover, the method has shown promise for evaluating seroconversion in vaccinated animals, thereby contributing to better vaccination strategies and herd management practices.

## MATERIALS AND METHODS

### Virus and cells

PCV2b was presented by the Animal Immune Engineering Institute of Jiangsu Academy of Agricultural Sciences. PK-15 cells were cultured in minimum essential medium (MEM) supplemented with 10% vol/vol newborn bovine serum (NBS) (C04001-500, Viva Cell Biosciences), 100 U/mL penicillin, and 100 µg/mL streptomycin at 37°C in a 5% CO_2_ atmosphere. The PCV2 Cap protein was produced using the baculovirus expression system, a gift from Dr. Chenglong (Shanghai Veterinary Research Institute).

### Evolutionary tree analysis of PCV2d

The whole genome of the PCV2d virus stored in the laboratory was analyzed by Mega software.

### Rescue of recombinant virus PCV2d

Due to the low titer of the PCV2d virus stored in the laboratory, which is insufficient to meet experimental requirements, we conducted a virus rescue. The plasmid pLB-PCV2d, containing the complete genomic sequences of the virus PCV2d strains, was synthesized by Genewiz, China. Subsequently, the plasmid pLB-PCV2d was excised using *Eco*R I (New England Biolabs, USA) digestion, resulting in the production of the complete PCV2d virus genomic sequences. The digested PCV2d genomic sequence products were separated by 1% agarose gel electrophoresis. The purified complete genomic fragments of PCV2d were ligated in the presence of T4 DNA ligase (TaKaRa, Japan) at 37°C for a brief duration of 10 minutes, promoting the generation of tandem dimers (56). These tandem dimers were purified using the QIAquick PCR purification kit (Qiagen, Germany) and subsequently cloned into the pBluescript SK (pSK) vector. After DNA sequencing, the recombinant plasmids pSK-PCV2d, which contain the tandem dimers of the PCV2d genome, were transfected into PK-15 cells (80%–90% confluency) in each well of a six-well plate utilizing FuGENE HD (Promega, USA) according to the manufacturer’s instructions. After a 6 hour incubation at 37°C, 2 mL of MEM containing 2% NBS and 3 mmol/L D-glucopyranose were added to each well. At 72 hours post-transfection, the cells underwent three freeze-thaw cycles, and the resultant supernatant was collected as the P0 generation virus. The P0 generation of recombinant porcine circovirus 2d (rPCV2d) was passaged to PK-15 cells for 16 successive generations.

### Production of monoclonal antibody against PCV2d Cap protein

A cohort of five 6-week-old female BALB/c mice underwent subcutaneous immunization with the rescued virus rPCV2d, receiving an initial dose of 100 µL per mouse. The mice were immunized a total of four times, with each immunization occurring 14 days apart to ensure full activation of their immune systems. Three days after the final immunization, the mice were euthanized, and polyethylene glycol was used to fuse the spleen cells with SP2/0 cells. Following 7 days of selection in the Hypoxanthine-Aminopterin-Thymidine (HAT) medium, hybridoma colonies were assessed for mAb 3G5 production and serially diluted to obtain single-cell clones. The mAb 3G5 was purified using a protein A gravity-flow column kit (Biodragon Biotechnology, China) for further characterization, resulting in a concentration of 2.5 mg/mL. The purified mAb 3G5 was labeled with HRP (Biodragon Biotechnology, China) to establish a blocking ELISA for the detection of PCV2 antibodies.

### SDS-PAGE analysis

Purified mAb 3G5 was mixed with 5× loading buffer (Beyotime Biotechnology, China) and denatured for 10 minutes at 100°C. Subsequently, 10 µL samples were resolved on 12.5% SDS-PAGE gel. The protein was electrophoresed for 120 minutes at 80 V using a BioRad Mini-Protean Tetra system, and the gel was stained with Coomassie Brilliant Blue for 1 hour at room temperature.

### Western blot

The ability of mAb 3G5 to bind to the PCV2 was evaluated using Western blot analysis. PK-15 cells were seeded in six-well plates with MEM enriched with 10% NBS and were incubated for 12 hours at 37°C. The PK-15 cells were subsequently infected with virus PCV2b and rescued virus rPCV2d at a multiplicity of infection (MOI) of 0.5 for 2 hours at 37°C in a 5% CO_2_ atmosphere. Post-infection, the cells were washed three times with phosphate-buffered saline after the removal of the supernatant, and then cultured at 37°C in fresh MEM supplemented with 2% NBS and 3 mmol/L D-glucopyranose. After 48 hours, the supernatants were discarded, and the cells were lysed using Radio-Immunoprecipitation Assay (RIPA) lysis buffer (Sangon Biotech, China), followed by the addition of a loading buffer to prepare the samples. The samples underwent denaturation for 10 minutes at a temperature of 100°C and were separated via 12.5% SDS-PAGE gels. After electrophoresis, the gels were transferred to a nitrocellulose (NC) membrane, which were then blocked for 2 hours in tris-buffered saline containing 5% (wt/vol) non-fat dry milk. The NC membrane was incubated overnight at 4°C with mAb 3G5 diluted 1:5,000 as the primary antibody, and afterward, it was incubated for 1 hour at room temperature with the corresponding HRP-conjugated affinipure goat anti-mouse IgG (H + L) secondary antibody (Proteintech, China). The binding of the HRP antibody was detected using a standard enhanced chemiluminescence (ECL) kit (NCM Biotech, China).

### IFA

To determine whether mAb 3G5 could bind to the Cap protein of PCV2 in rPCV2d-infected PK-15 cells, PK-15 cells were cultured in 24-well plates until they reached 80% confluence and subsequently infected with virus PCV2b and rescued virus rPCV2d (MOI = 0.5). Following an adsorption period of 2 hours at 37°C, the culture medium was exchanged for fresh MEM supplemented with 2% NBS and 3 mmol/L D-glucopyranose. At 48 hours following infection, the PK-15 cells that had been infected were fixed for 10 minutes at room temperature using 4% paraformaldehyde. Subsequently, these virus-infected cells underwent permeabilization with 0.5% Triton X-100 for 10 minutes and were then blocked with 3% Bovine Serum Albumin (BSA) in Phosphate Buffered Saline Tween-20 (PBST) for 30 minutes at 37°C. Finally, the cells were incubated with mAb 3G5 (diluted 1:5,000) at 37°C for a duration of 1 hour. After three washes, Alexa Flour 488-conjugated goat anti-mouse IgG (H + L) (ThermoFisher, USA) was applied as the secondary antibody and incubated at 37°C for 1 hour in the dark. Finally, the cells were observed using a Nikon fluorescent microscope.

### Establishment of the blocking ELISA

The checkerboard titration technique was employed to identify the ideal concentration for coating with the PCV2 Cap protein antigen and to establish the working concentration of the mAb 3G5. In this study, the purified PCV2 Cap protein was diluted to 0.01 µg/mL, 0.025 µg/mL, 0.05µg/mL, 0.1µg/mL, 0.5µg/mL, 1.0 µg/mL, 2.0 µg/mL, 3.0 µg/mL, 4.0 µg/mL, 5.0 µg/mL, and 6 µg/mL, which were then introduced into the wells of a 96-well ELISA plate (Corning Inc., Kennebunk, ME, USA) and incubated for 12 hours at 4°C. After washing the plates three times with PBST, they were blocked with a 5% skim milk solution diluted in PBST for an additional 12 hours at 4°C. After another three washing rounds with PBST, 100 µL of both positive and negative reference pig serum, each diluted 1:1, was added to the designated wells and left to incubate overnight at 4°C. After this step, the wells were washed again, and 100 µL of mAb 3G5 with HRP conjugated, diluted at 1:1,000, 1:2,000, and 1:5,000, was added and incubated at 37°C for 1 hour. Finally, 100 µL of the chromogenic substrate 3,3′,5,5′-tetramethylbenzidine was added to each well and allowed to develop for 15 minutes at room temperature in the dark. The addition of 50 µL of 2 M H_2_SO_4_ to each well halted the reaction, and an ELISA microplate reader was used to measure the optical density (OD) of the samples at 450 nm (Biotek, USA). The results were converted to the percentage inhibition (PI) using the formula: PI (%) = (1 − [OD_450 nm_ value of test serum sample/OD_450 nm_ value of negative control serum]) × 100%.

### Specificity and sensitivity analysis of the blocking ELISA

In order to assess the specificity of the blocking ELISA, five antibody-positive serum samples against various swine viruses (PCV2, ASFV, PRRSV, PEDV, and PRV) were tested. The sensitivity of the blocking ELISA method was assessed by conducting a twofold serial dilution of 11 serum samples that tested positive for PCV2. These 11 pig serum samples were collected from pigs immunized with PCV2 baculovirus vector vaccine (Boehringer Ingelheim Animal Health, USA) and kept in our laboratory. We have utilized a commercial indirect ELISA kit to assess the PCV2 antibody levels in the 11 serum samples, all of which yielded positive results (data not shown). The dilutions ranged from a ratio of 1:1 to 1:256, allowing for a comprehensive evaluation of the assay’s detection capabilities.

### Repeatability and reproducibility assessment of the blocking ELISA

The repeatability of the blocking ELISA was assessed by calculating the CV for both inter-batch and intra-batch variations of the 11 serum samples’ PI values (comprising seven positives and three negatives). To assess the inter-assay CV, each serum sample underwent testing on three distinct plates. This approach allows for a comprehensive evaluation of the variability between different assay runs. Additionally, to establish the intra-assay coefficient of variation, three replicates of each sample were analyzed on the same plate.

### Comparison of the blocking ELISA and commercial ELISA kit

To evaluate the consistency between the newly developed blocking ELISA and a commercial indirect ELISA kit, a comprehensive analysis was conducted using 402 clinical serum samples collected from large-scale agricultural farms. Both the blocking ELISA and the commercial indirect ELISA were utilized to test these samples. The relative coincidence rate (kappa value) between the two assays was determined using SPSS software, which facilitated the analysis of the results. In the context of this assessment, a higher kappa value was indicative of a superior coincidence rate. Specifically, a kappa value within the range of 0–0.4 pointed to poor consistency between the blocking ELISA and the commercial indirect ELISA, while a kappa value of 0.75 or greater was indicative of a high level of consistency between the two testing methods.
